# Silent Scream: A Phenomenological Study of Female Nurses' Experiences of Workplace Bullying

**DOI:** 10.1111/jep.70486

**Published:** 2026-06-23

**Authors:** Hava Salik, Ahmet Salik

**Affiliations:** ^1^ Department of Health Management, Faculty of Economics and Administrative Sciences Hakkari University Hakkari Türkiye; ^2^ Van Provincial Health Directorate Edremit No. 3 Family Health Center Van Türkiye

**Keywords:** nurses, qualitative study, workplace bullying

## Abstract

**Aim:**

This descriptive phenomenological study explored the lived experiences of workplace bullying among female nurses in Türkiye.

**Design:**

A descriptive phenomenological study.

**Methods:**

Between September 2024 and August 2025, online semi‐structured in‐depth interviews were conducted with 16 female nurses working in public, private and university hospitals across different regions of Türkiye.

**Results:**

Four main themes emerged: ‘Mobbing’, ‘Effects of Mobbing’, ‘Coping Methods’ and ‘Suggested Solutions’. Workplace bullying was found to be a multidimensional organisational problem that profoundly impairs nurses’ emotional, physical and professional well‐being.

**Conclusion:**

Workplace bullying is a multidimensional organisational problem that negatively affects nurses’ emotional, physical and professional well‐being. Preventing workplace bullying requires not only individual awareness but also institutional policies with enforcement power and strong managerial support mechanisms.

## 1 Introduction

Workplace bullying is considered a serious psychosocial stressor that can trigger mental health issues such as anxiety, depression and post‐traumatic stress in nurses or exacerbate the severity of these issues [1, 2]. Nurses frequently face bullying in the form of unfair criticism, exclusion, being ignored, verbal abuse and authoritarian pressure [3, 4], which negatively affects their psychological and physical well‐being and the quality of patient care [5, 6]. Female nurses may be more vulnerable to such behaviour due to gender roles, power relations and hierarchical dynamics in the workplace [7, 8]. Although ‘mobbing’ and ‘bullying’ are often used interchangeably in the international literature, nuanced differences exist between them [9]. In Türkiye, the term ‘mobbing’ (also referred to as psychological harassment or workplace bullying) is predominant and describes a specific form of systematic psychological aggression in the workplace [10].

This study was guided by the following research question:

What are the lived experiences of female nurses subjected to workplace mobbing in Turkish hospital settings?

## 2 Background

The Ministry of Labor and Social Security's Mobbing Information Guide defines workplace mobbing as malicious behaviours that continue over a certain period of time and harm the victim's personality, professional life, social relationships, or health [11, 12] suggested that these behaviours must continue for at least 6 months, be repeated several times a month, and involve a clear power imbalance between the parties. Organizational causes include negative working conditions, communication problems, uncertainty in tasks, social isolation and deficiencies in leadership behaviour [2].

The concept of bullying in nursing encompasses hostile behaviours directed physically or psychologically by colleagues, physicians, managers, patients/patient relatives and other healthcare workers [13, 14, 15]. Aydın and Öcel's study found that female nurses were exposed to more bullying than male nurses [16].

As the mobbing process continues, nurses experience increased burnout, anxiety, depression and intention to leave their jobs; meanwhile, the quality of patient care is negatively affected [5, 6]. Nurses are a group with a high tendency to encounter bullying behaviour due to their close relationship with patients and their families, the fact that most members of the profession are women, the lack of homogeneity within the profession due to different educational levels, and their dependent functions based on physician orders [13]. Despite growing awareness, workplace bullying among nurses—particularly female nurses—remains under‐explored in the Turkish context, especially from a gendered and phenomenological perspective. Using a descriptive phenomenological approach, this study aimed to deeply illuminate these experiences and their implications for mental health nursing practice. In addition to the literature, the researcher's field observations during professional interactions with nurses have revealed that bullying behaviours are frequently normalised within organisational cultures and that this situation is underreported. These observations have shown that hierarchical pressure, communication breakdowns and a culture of silence contribute to the persistence of bullying in the workplace. This contextual insight further supports the need for a deep, phenomenological examination of the experiences of female nurses.

### 2.1 Design

In this study, a descriptive phenomenological design was used to explore the experiences of female nurses exposed to workplace bullying. This design was chosen because it provides flexibility in defining and understanding the phenomenon from the participants’ perspective, enabling the collection of rich, in‐depth data and the detailed portrayal of lived experiences [17]. The phenomenological approach seeks to reveal the essence and meaning of individuals’ lived experiences of a particular phenomenon [18, 19]. The analysis was guided by a phenomenological theoretical lens, which prioritises the subjective experiences of participants as the foundation for meaning‐making in understanding workplace bullying.

### 2.2 Participants

Criterion sampling, one of the purposeful sampling methods, was used to determine the study group for the research. Criterion sampling ensures that the sample consists of individuals, events, objects, or situations that possess the characteristics defined in relation to the problem [20, 21]. The inclusion criteria were defined as being a female nurse, reporting having experienced bullying in the last 12 months, and volunteering to participate. All nurses who met the criteria were invited to the interview. Social media platforms such as WhatsApp, Twitter, Facebook and Instagram, as well as various announcement channels, were used to reach the nurses. As a result of the call, 21 applications were received. During the interviews, data saturation was reached by monitoring recurring codes between the 14th and 16th interviews for thematic saturation, and data collection was terminated at this stage [17].

The research process was conducted based on the COREQ (Consolidated Criteria for Reporting Qualitative Research) checklist [22].

### 2.3 Data Collection

The research was conducted between September 2024 and August 2025 with female nurses working in public, private, and university hospitals in different regions of Türkiye. Each participant was interviewed once, and each interview lasted an average of 30–45 min. Audio recordings were made with the nurses’ consent.

The semi‐structured interview guide was developed by the first author (H.S.), informed by prior literature [23, 24, 25], and refined based on expert review (ÖU, MŞ, ZKŞ). The full English version of the interview guide is provided as Additional file 1. All interviews were conducted online by the first author (H.S.), an experienced qualitative researcher [23, 24, 25]. The semi‐structured interview questions used in this study are presented in Table 1.

**Table 1:** Interview guide.

The demographic characteristics of nurses were obtained through questions such as gender, age, marital status, number of children, professional experience, length of service in the unit and economic status
*The semi‐structured interview questions were as follows:*
1. How would you describe your experiences of mobbing? How did this situation affect the general atmosphere and communication styles at your workplace?
2. What emotional and psychological effects did you experience when you were subjected to mobbing? How did these effects reflect on your work performance?
3. Were the support mechanisms (institutional, social, individual) you resorted to during the mobbing process sufficient? How did this support affect your experience?
4. How do you assess the relationship between bullying and organizational silence? How did this situation affect your tendency to remain silent?
5. How did the experiences you had after bullying affect your overall satisfaction and motivation at work?

*Note:* The interview guide is also available in full in Additional file 1.

### 2.4 Data Analysis

Data were analysed using Colaizzi's (1978) seven‐step descriptive phenomenological method: (1) familiarisation, (2) extraction of significant statements, (3) formulation of meanings, (4) clustering into themes, (5) exhaustive description, (6) identification of fundamental structure and (7) member checking [26]. All interviews were transcribed verbatim and the accuracy of the transcripts was checked against the audio recordings. The researcher read each transcript several times to get a sense of the whole and to grasp what the participants wished to express. The transcripts were then systematically analysed line by line and significant utterances expressing the nurses’ mobbing experiences were identified. At this stage, the researcher performed preliminary coding for analysis by identifying important emphases in the participants’ emotional expressions, reactions and experiences. The statements identified were interpreted in a way that stayed true to the participants’ stories, uncovering their deeper meanings. This process helped distil their experiences into essential insights that reflect the realities faced by the nurses. Meaningful statements and formulated meanings were grouped according to similar characteristics and collected under sub‐themes and themes. Relationships between the emerging themes were defined, and their contents were detailed in a way that reflected the depth and diversity of the nurses' experiences. The themes obtained were brought together in a way that reflected the essence of the phenomenon and formed a comprehensive description. All themes were synthesised to explain the basic structure and core meaning of the phenomenon.

Thematic summaries developed to increase the reliability of the findings were shared with the participants. Minor corrections were made based on feedback from two participants; no feedback was received from the others. Data were managed and coded using MAXQDA 2022 qualitative analysis software [27]. The software enabled systematic coding of data, code‐comment matching and visual tracking of thematic classifications. The coding process was initially performed by the researcher, then the codes and themes were independently reviewed by three experts (MŞ, ÖU, ZKŞ). Discussions continued until consensus was reached, and the consensus‐based analysis was completed. This reflective approach ensured that the researcher consciously managed their interaction with the data and that interpretations remained faithful to the participants’ statements. Recognising the researcher's positionality and documenting the process increased transparency and the scientific trustworthiness of the analysis. Bracketing was employed to ensure that the researcher's preconceptions did not influence the interpretation of the data. The researcher maintained reflexive awareness throughout the analysis process and systematically documented personal assumptions.

## 3 Trustworthiness and Theoretical Framework

### 3.1 Trustworthiness

The findings were subjected to a variety of confirmatory procedures to verify their credibility, dependability, confirmability and transferability [28]. These were: expert cheques on the codes and themes, reflective journals maintained by the researcher throughout, and peer‐debriefing to verify interpretations.

## 4 Theoretical Framework

The research is grounded in phenomenological methodology, which seeks to achieve understanding of human experiences through exploration [29]. This aligns with the philosophy of psychiatry and mental health nursing wherein core values, including empathy, reflexive understanding, non‐instrumentalism and non‐paternalism in person‐centred care are integral.

## 5 Findings

Findings regarding the sociodemographic data of nurses exposed to workplace bullying are presented in Table 2.

**Table 2:** Descriptive characteristics of nurses.

Participant	Gender	Age	Marital status	Number of children	Years of professional experience	Years in current unit	Perceived economic status
K1	Female	29	Married	1	7	3	Poor
K2	Female	53	Single	3	35	16	Moderate
K3	Female	41	Married	1	18	2	Good
K4	Female	31	Married	2	15	2	Poor
K5	Female	25	Single	0	5	5	Poor
K6	Female	26	Married	0	4	2	Moderate
K7	Female	45	Single	1	22	2	Poor
K8	Female	49	Married	1	31	21	Poor
K9	Female	49	Married	2	32	17	Poor
K10	Female	30	Single	0	8	3	Moderate
K11	Female	47	Married	2	30	2	Good
K12	Female	31	Married	0	14	2	Moderate
K13	Female	47	Married	2	20	5	Moderate
K14	Female	23	Single	0	2	2	Moderate
K15	Female	43	Married	2	23	17	Moderate
K16	Female	27	Single	0	4	2	Moderate

All nurses participating in the study are female. Among those with children, the number of children ranges from 1 to 3. Their professional experience ranges from 2 to 35 years, with an average of 17.5 years. Their length of service in their current unit ranges from 2 to 21 years, with an average of 5.3 years. Their perception of economic status is mostly ‘moderate’. However, those who rated it as ‘poor’ constitute a significant group, while the number of those who rated it as ‘good’ is limited (Table 2).

Four themes were identified in the analysis of the data: mobbing, the effects of mobbing, coping methods and suggested solutions (Table 3).

**Table 3:** Themes, sub‐themes and codes related to the experiences of female nurses.

Theme	Subtheme	Code	*n* *
Mobbing	Definition	Intimidation policies	8
		Psychological violence	6
		Coercion and insistence behaviours	6
		Oppression	4
		Being ignored	6
	Behaviours	Not greeting	8
		Assigning all tasks	11
		Unjust accusations	3
		Disapproval of work performance	3
		Verbal humiliation	6
		Constant interruption while speaking	3
	Reasonsfor Exposure	Power display and authority assertion (senior–junior hierarchy, isolation)	5
		Communication problems	10
		Personality traits	3
Effects of Mobbing	Emotional	Anger	16
		Sadness	13
		Feelings of worthlessness	13
		Anxiety	10
		Loneliness	4
		Burnout	3
	Physical	Fatigue	13
		Sleep problems	5
		Teeth clenching	4
		Headache	4
	Professional	Decreased motivation	16
		Increased malpractice risk	8
		Reduced compassion	8
		Intention to resign or retire	7
Coping Strategies	Social	Sharing with family	10
		Sharing with colleagues	8
	Professional	Seeking professional support	3
		Using medication	6
	Individual	Engaging in activities (reading, watching movies, sports, etc.)	10
		Using social media	10
	Behavioural	Focusing on work	8
		Constant complaining	4
		Ignoring the situation	5
	Cognitive	Normalising the situation	8
		Trying not to think about it	3
Proposed Solutions	Recommendations	Enhancing professional status	5
		Strengthening professional organizations	8
		Improving working conditions	8
		Managerial support	5
		Legal sanctions and enforcement	16

* *n* folded.

## 6 Theme 1: Mobbing

Three sub‐themes have been identified under the theme of mobbing: ‘Definition’, ‘Behaviours’ and ‘Reasons for Exposure’. This theme reveals the mobbing experiences of nurses in the workplace and the psychosocial dimensions of these experiences.

Some of the nurses’ statements are as follows:

They are implementing a policy of intimidation, trying to remove him from that unit in some way and push her out of their domain…

(K7)

Mobbing is pressure in your environment, either physical or psychological. Intense pressure…

(K15)

I think mobbing is pressure, forcing things on someone. Being forced to do things you don't want to do…

(K6)

You know the truth, but they insist you're wrong. Even though you say it's wrong, the other side refuses to accept your truth just to satisfy their ego and pressures you to do the wrong thing…

(K15)

Isolation, within the institution. And feeling incompetent and worthless…

(K3)

## 7 Theme 2: Effects of Bullying

Within the theme of the effects of bullying, three sub‐themes have been identified: ‘Emotional’, ‘Physical’ and ‘Professional’. The nurses’ statements reveal the multidimensional effects of bullying on individuals.

Some of the nurses’ statements are as follows:

All of this I've experienced after the mobbing has seriously lowered my overall satisfaction at work. My motivation too…

(K5)

I'll quit today. That's it. If my living conditions weren't already set, I would definitely try to find another alternative…

(K4)

I've grown so cold towards the service that I've grown cold towards working. I've grown cold toward the place where I used to work happily…

(K9)

When you start a job, they teach you certain things, but then you come and can't see them put into practice. It's like, ‘This is such a valuable profession, it's still a valuable profession to work here,’ but it doesn't kill the profession itself. It's just that when you come here, you can't do the job…

(K16)

I get angry. I've started clenching my teeth really badly…

(K4)

My nerves are getting frayed, my nerves are getting frayed. I think it would be a much more workable environment if everyone did their job properly in the emergency room…

(K8)

I get extremely irritated. For example, my brain feels like it's too small for my skull, I get hot flashes, I get tachycardia…

(K15)

You lose touch with reality and shape yourself according to what other people say. You shape your own work according to what they say…

(K5)

It makes you feel bad. It makes you feel worthless…

(K1)

Stress, tension, and anxiety, which of course affect work performance…

(K2)

Very long working hours, frequent shifts, not having time for social life, the money you earn becoming worthless.

(K 5)

My sleep pattern was disrupted and I couldn't sleep at night. I kept waking up, wondering if I was late, if it had happened, counting the minutes at work.

(K 16)

## 8 Theme 3: Coping Methods

Five sub‐themes have been identified under the theme of Coping Methods: ‘Social’, ‘Professional’, ‘Individual’, ‘Behavioural’ and ‘Cognitive’. This theme reveals the coping strategies developed by nurses against workplace bullying and the personal and social dimensions of these strategies.

Some of the nurses’ statements:

I watch movies. I watch TV. I take care of my children…

(K4)

Then I go to the gym, I work out, I work out. Then I walk for hours and hours. Then I go home. I cook. I spend time with my family…

(K15)

I ignored their bullying. I intervened when necessary, when it turned physical…

(K3)

I try not to think about it and don't dwell on it…

(K4)

Of course, it provides short‐term relief. After all, you go home, share it with your spouse, and yes, you feel relieved at that moment, but when you come back, the same things happen again. It's a vicious cycle, the same things happen again, the same cycle continues like this…

(K1)

## 9 Theme 4: Proposed Solutions

A sub‐theme titled ‘Recommendations’ has been identified within the Proposed Solutions theme. This theme highlights the solutions developed by nurses in combating mobbing and the strategies they propose.

Some of the nurses’ statements are as follows:

I wish everyone wouldn't stay silent about bullying. I mean, if we had more support mechanisms, we could deal with it seriously and officially instead of just complaining to someone. And there should be consequences.

(K5)

If there were consequences for bullying, exactly, if there were serious consequences, or if bullying could be proven with more concrete evidence…

(K5)

Everyone wants to work happily. Everyone wants to work positively. Everyone wants to work under better conditions…

(K10)

When we didn't speak up, they came down on us even harder. Then they changed our positions…

(K12)

When someone does something, they think, ‘Well, the other side isn't speaking up’. Since the environment isn't speaking up either, that person feels entitled, they get away with it, and it grows even more with the silence…

(K14)

## 10 Discussion

Nurses have described workplace bullying as a mix of ‘psychological pressure, social isolation and authoritarianism’. These insights align with definitions found in Türkiye and the broader international literature [5, 7]. Workload pressure, unfair criticism and verbal abuse were the most common types of mobbing. Mobbing is triggered by several factors, with the important ones being communication failures, power struggles and inflexible hierarchical structures, and is increasingly viewed as not just an interpersonal struggle but a symptom of dysfunction within organizational processes [30, 31]. Nurses often find themselves facing social isolation, feeling undervalued, and dealing with ongoing criticism from their colleagues or supervisors [5, 7, 8, 32]. Yet bullying has also been linked to organizational culture, management style and business policies. Hierarchical configurations that fail to encourage transparent communication and collaboration can also foster the emergence of bullying behaviours among colleagues [30, 31]. The bullying behaviours experienced by nurses are not only an individual problem but are also closely related to organizational communication deficiencies, power relations and managerial approaches. In the study conducted by Güngör Tavşanlı and colleagues, it was found that as nurses’ workplace bullying scores increased, their quality of life decreased in subdimensions such as physical function, physical role limitations, vitality/energy, pain and emotional role limitations [14]. The analyses revealed serious emotional consequences such as widespread anger, sadness and anxiety. Physically, participants reported psychosomatic symptoms such as exhaustion, sleep problems, and headaches. From a professional perspective, mobbing has created a situation that directly threatens patient safety by leading to negative outcomes such as loss of motivation, a significant decrease in job satisfaction, and an increased risk of medical errors [5, 6]. Individuals exposed to mobbing often feel lonely, helpless and worthless at work; this seriously undermines their motivation, leading to withdrawal from work and even the desire to change jobs [5, 7]. Mobbing not only harms nurses’ mental and physical health but also directly affects the quality of patient care [3, 5]. Shorey and Wong qualitative systematic review confirms that bullying causes physical (sleep disorders, headaches), emotional (anger, sadness, feelings of worthlessness, anxiety) and psychological (burnout, low self‐esteem) distress in victims and negatively affects work performance [15]. At the same time, communication breakdowns increase, team cohesion weakens and consequently, the effectiveness of care processes declines [4, 6]. In the study, nurses stated that they used various strategies to cope with mobbing. Participants indicated that they tried to reduce the negative effects of mobbing through social (family and colleague support), individual (engaging in activities, using social media), professional (taking medication, receiving psychological support), behavioural (focusing on work, complaining, ignoring) and cognitive (normalising, trying not to think about it) approaches. These findings indicate that nurses mostly develop coping strategies through individual efforts, but most of these strategies provide only short‐term relief. This situation is consistent with findings obtained in other care settings. In these settings, when faced with systemic difficulties, employees mostly resort to their personal resilience and informal support sources; this situation clearly demonstrates the need for structured organizational support [33]. These programs should teach nurses what bullying entails, how it manifests, and how to respond effectively [3, 34]. Such structured training is thought to help nurses strengthen psychological resilience, manage stress more effectively, and develop protective attitudes against workplace difficulties [35, 36]. As Shorey and Wong pointed out in their study, nurses employ a range of individual and social coping mechanisms to deal with bullying, including obtaining support from family and friends, disregarding the matter, eluding it, or concentrating on work. Nevertheless, these practices do not always lead to a permanent resolution [15].

Nurses in this study also emphasised that legally enforceable regulations should be established to prevent mobbing and mitigate its effects, that managers should adopt supportive attitudes, that professional organizations should be strengthened, and that working conditions should be improved. These recommendations underline the need for systemic change rather than individual efforts in combating mobbing. Preventing workplace bullying is directly related to organizational culture, leadership style and communication policies. Studies show that bullying is more prevalent in organizations with authoritarian leaders and poor communication [37, 38], but the magnitude of these behaviours is remarkably reduced in cultures that foster cooperation, collegiality and fairness. Thus, building a culture of open communication, ensuring administrative justice and enhancing team cohesion are important in preventing bullying and improving nurses job satisfaction [39]. The person‐centred care model highlights that trust stability and openness are key elements of a supportive work environment. In such a setting, employees feel valued, can express themselves freely, and are empowered to make meaningful contributions to the organization's activities. Psychiatric nurses possess unique skills to recognise and intervene early in the mental consequences of mobbing. Based on these findings, several mental health interventions can be proposed for the workplace. To start, introducing regular mental health screenings for nurses in high‐stress settings could help catch psychological distress linked to bullying early on. Additionally, providing psychoeducational programs that focus on developing coping skills and resilience can empower nurses to handle the emotional toll of mobbing more effectively. Creating peer support groups, led by mental health professionals, would also offer a safe environment for nurses to share their experiences and combat stigma. Lastly, having strict and consistently enforced anti‐bullying policies, backed by a zero‐tolerance approach at the institutional level, is crucial. These interventions are not optional; rather, they are essential ethical responsibilities and key competency areas for psychiatric and mental health nurses in their clinical, administrative and educational roles.

It is important to develop clear definitions and sanctions at the institutional and national levels to protect the well‐being of nurses and maintain high quality care. Training programs for nurses on how to manage bullying have been reported to be successful and evidence suggests a decrease in the frequency of bullying per week [13, 34]. This result underscores the significance of awareness training and institutional policy, which are best practice solutions. When viewed from the perspective of psychiatric nursing, these results inform possible needs for organised psychological support, resilience‐enhancement strategies and counselling for nurses who may experience mobbing. Psychiatric/mental health nurses have a central role in the development and delivery of workplace mental health promotion initiatives to mitigate the psychosocial impact of bullying. The incorporation of such strategies into clinical care not only improves the psychological well‐being of nurses but also aids in building safer, more supportive environments of care.

## 11 Limitations

The findings of this study are based solely on the experiences of a limited number of female nurses working in public, private, and university hospitals across different regions of Türkiye, which may limit the transferability of the results. Since the entire sample consisted of female nurses, the findings do not reflect gender diversity. A potential limitation of this study relates to researcher bias inherent in qualitative research. To minimise this, bracketing was systematically applied throughout the research process. The researcher consciously set aside prior assumptions and maintained reflexive notes during both data collection and analysis. In addition, peer debriefing and independent coding by multiple researchers were employed to enhance credibility and reduce interpretive bias. Despite these measures, the subjective nature of phenomenological interpretation may still influence the findings. Future research should involve more diverse samples, including different care provider profiles and institutional settings. To gain a clearer understanding of gender dynamics in workplace bullying, further studies including male nurses and more heterogeneous participant groups are recommended.

## 12 Conclusion

This study delved into the real‐life experiences of female nurses who faced workplace bullying, revealing that its impact is wide‐ranging, affecting both their personal lives and professional careers. The results highlight the importance of a multi‐level approach to bullying prevention that involves institutional policies, administrative support and legal regulations that safeguard nurses’ rights. Nurses participating in this study emphasised the need for legally binding measures, a more supportive attitude from management, stronger professional organizations and better working conditions. The results indicate that mobbing is not simply an interpersonal issue, but rather an organizational issue embedded in organizational culture and leadership style. To create lasting change, reliable, consistent and open institutional systems must be established, similar to those emphasised in person‐centred care models. In conclusion, the result of this research offers a number of practical implications for psychiatric and mental health nursing. Mental health screenings, psychoeducation and peer support systems in the healthcare environment may also empower nurses, improve coping and foster psychologically safe work environments.

## Author Contributions

**Hava Salik:** study conception and design, data collection, data analysis and interpretation, manuscript preparation and critical revision. **Ahmet Salik:** contribution to the introduction, discussion and conclusion sections, manuscript review and editing, critical revision.

## Funding

The author has nothing to report.

## Ethics Statement

This research has been approved in accordance with the decision of the Scientific Research and Publication Ethics Committee of Hakkari University dated 17.09.2024 and numbered 2024/154‐1.

## Consent

Informed consent was obtained from the nurses who agreed to participate in the research. This study was conducted in accordance with the principles of the Declaration of Helsinki.

## Conflicts of Interest

The authors declare no conflicts of interest.

## Data Availability

The data that support the findings of this study are available on request from the corresponding author. The data are not publicly available due to privacy or ethical restrictions.
